# Inhibition of StearoylCoA Desaturase-1 Inactivates Acetyl-CoA Carboxylase and Impairs Proliferation in Cancer Cells: Role of AMPK

**DOI:** 10.1371/journal.pone.0006812

**Published:** 2009-08-27

**Authors:** Natalia Scaglia, Jeffrey W. Chisholm, R. Ariel Igal

**Affiliations:** 1 Department of Nutritional Sciences and Rutgers Center for Lipid Research, Rutgers, the State University of New Jersey, New Brunswick, New Jersey, United States of America; 2 Biology, Gilead Sciences, Inc., Palo Alto, California, United States of America; National Institutes of Health (NIH)/National Institute of Environmental Health Sciences (NIEHS), United States of America

## Abstract

Cancer cells activate the biosynthesis of saturated fatty acids (SFA) and monounsaturated fatty acids (MUFA) in order to sustain an increasing demand for phospholipids with appropriate acyl composition during cell replication. We have previously shown that a stable knockdown of stearoyl-CoA desaturase 1 (SCD1), the main Δ9-desaturase that converts SFA into MUFA, in cancer cells decreases the rate of lipogenesis, reduces proliferation and in vitro invasiveness, and dramatically impairs tumor formation and growth. Here we report that pharmacological inhibition of SCD1 with a novel small molecule in cancer cells promoted the activation of AMP-activated kinase (AMPK) and the subsequent reduction of acetylCoA carboxylase activity, with a concomitant inhibition of glucose-mediated lipogenesis. The pharmacological inhibition of AMPK further decreased proliferation of SCD1-depleted cells, whereas AMPK activation restored proliferation to control levels. Addition of supraphysiological concentrations of glucose or pyruvate, the end product of glycolysis, did not reverse the low proliferation rate of SCD1-ablated cancer cells. Our data suggest that cancer cells require active SCD1 to control the rate of glucose-mediated lipogenesis, and that when SCD1 activity is impaired cells downregulate SFA synthesis via AMPK-mediated inactivation of acetyl-CoA carboxylase, thus preventing the harmful effects of SFA accumulation.

## Introduction

Cancer cells display a radically modified metabolism that promotes their continuous proliferation. As part of the metabolic shift towards macromolecular synthesis to support cell replication, cancer cells activate the biosynthesis of saturated fatty acids (SFA) and monounsaturated fatty acids (MUFA) to sustain an increasing demand for phospholipids of appropriate acyl composition for membrane biogenesis. Thus, several critical enzymes involved in de novo fatty acid synthesis have been shown to be overexpressed in malignant cells: ATP-citrate lyase, required for the production of cytosolic acetylCoA [1], acetylCoA carboxylase (ACC), the enzyme that catalyzes the synthesis of malonylCoA, the first committed step in the synthesis of fatty acids [2], [3], and fatty acid synthase (FAS), which synthesizes SFA [2]. The importance of fatty acid synthesis for cancer cell proliferation and survival is highlighted by the fact that the inhibition of any of these enzymes leads to a halt in cell proliferation and increased cell death [4]–[9]. However, in spite of the overactivation of the tandem of biosynthetic enzymes that ultimately renders SFA, abundant amounts of MUFA are typically found in cancer cells [10]–[13], suggesting that the biosynthesis of MUFA is required to ensure cancer cell proliferation and survival.

Mammalian stearoylCoA desaturases (SCD) are microsomal enzymes that catalyze the Δ9-desaturation of saturated acylCoAs to form monounsaturated derivatives [14]. The expression of SCD1, the main SCD isoform, is increased in several human cancers, chemically induced tumors, as well as in oncogene-transformed cells [1], [13], [15]–[18]. We have shown that SCD1 modulates not only the content of MUFA in cancer cells, but also the overall process of lipogenesis [19]. Remarkably, the ablation of SCD1 expression reduces cancer cell proliferation and in vitro invasiveness, and dramatically impairs tumor formation and growth [19], [20]. We have also found that active SCD1 may be required for neoplastic cells to survive a lipotoxic stress since SCD1 knockdown increases basal apoptosis and sensitizes the cells to the cytotoxic effects of excess SFA [19]. SCD1 has also been identified from a siRNA library as a gene whose suppression impairs human cancer cell survival, further supporting a functional link between SCD1 and cancer cell growth [21]. Nevertheless, despite this growing body of information, the intricate mechanisms by which SCD1 concurrently modulates lipid metabolism and the biological features of cancer cells are not known.

The process of lipogenesis in mammalian cells is regulated by Akt and AMP-dependent protein kinase (AMPK), two major signaling proteins that control several critical biosynthetic and catabolic reactions. Akt is a powerful inducer of glucose-mediated lipogenesis in cancer cells, mainly regulating the activity and transcription of multiple enzymes of glycolysis and fatty acid synthesis [22], [23]. As part of a feedback loop, the activity of Akt is modulated by the levels of FAS and SCD1. It was observed that blockade of FAS activity and ablation of SCD1 expression decrease Akt phosphorylation and activity in cancer cells [20], [24]. In contrast, AMPK activation by phosphorylation promotes the downregulation of several lipogenic pathways and activates energy-supplying reactions such as fatty acid oxidation [25]. One major target of activated AMPK is ACC. Upon phosphorylation by AMPK, ACC activity is decreased resulting in the inhibition of de novo fatty acids synthesis [26]. The concomitant reduction of malonylCoA levels promotes the β-oxidation of fatty acids. SFA are also potent allosteric inhibitors of ACC, providing a negative feedback loop for the fatty acid biosynthesis [27]–[29]. We hypothesize that elevated SCD1, by converting SFA to MUFA, is able to maintain the pathway of fatty acid synthesis and lipogenesis fully activated. This condition favors cancer cell growth and proliferation, hence reducing SCD1 activity should impair these two biological processes.

Recently, several series of novel Δ9-desaturase selective small-molecule inhibitors have been published [30]–[32]. One of these SCD inhibitors, CVT-11127 (N-(2-(6-(3,4-dichlorobenzylamino)-2-(4-methoxyphenyl)-3-oxopyrido[2,3-b]pyrazin-4(3H)-yl)ethyl) acetamide), is a potent and specific inhibitor of rat microsomal and HepG2 cell Δ9-desaturation [30] and may be a potential valuable tool for studying the regulation of cellular metabolism and signaling pathways by SCD1 activity.

In our current studies, we show that the acute inhibition of SCD1 activity with CVT-11127, as well as the chronic deficiency of SCD1 by stable gene knockdown, significantly impairs de novo fatty acid synthesis from glucose in human lung carcinoma cells. We also report that pharmacological inhibition of SCD activity drastically reduced cellular proliferation in cancer cells, confirming that SCD1 activity is a crucial requirement for cancer cell growth. Furthermore, we observed that the blockade of SCD1 activated AMPK and inactivated ACC resulting in decreased lipogenesis. In experimental conditions that induce lipogenesis, such as stimulation of ACC with citrate and inhibition of AMPK, a further decrease in cellular proliferation was observed in SCD1-deficient cells. In contrast, the pharmacological activation of AMPK reversed cell proliferation to control levels. As a whole, our data suggest that the downregulation of fatty acid synthesis when SCD1 activity is low may be an adaptive safeguard mechanism to prevent the harmful effects of excess SFA when its conversion to MUFA is impaired. Moreover, these results highlight the importance of SCD1 in the regulation of neoplastic proliferation and metabolism.

## Materials and Methods

### Materials

A549 human lung adenocarcinoma cells and WS-1 human fibroblasts were obtained from the American Type Culture Collection (Rockville, MD, USA). H1299 human lung cancer cells and MCF-7 human breast cancer cells were generously provided by Dr C. S. Yang and Dr Wendie Cohick, Rutgers University, NJ, respectively. Dulbecco's modification of Eagle's medium (DMEM) with L-glutamine, MEM vitamin mixture and MEM nonessential amino acid solution were from Mediatech Cellgro (Manassas, VA, USA). Minimum Essential Medium (MEM) containing Earle's salts and L-glutamine, glucose free DMEM, phenol red free MEM, trypsin-EDTA solution and Lipofectamine™ 2000 transfection reagent were purchased from Invitrogen Corporation (Carlsbad, CA, USA). Heat-inactivated fetal bovine serum, crystal violet, protease and phosphatase inhibitor cocktail 2, fatty acid free bovine serum albumin, monoclonal anti β-actin antibody, AICAR, coenzyme A, ATP, NADH and dimethyl sulphoxide (DMSO) were from Sigma-Aldrich (St. Louis, MO, USA). Nitrocellulose membrane, HPLC grade solvents, phosphate-buffered solution without calcium and magnesium and other cell culture supplies were obtained from Thermo Fisher Scientific (Pittsburgh, PA, USA). Anti phospho-AMPKα (Thr172) and phospho-ACC (Ser79) antibodies were obtained from Cell Signaling Technology Inc (Danvers, MA, USA). HRP-conjugated anti mouse and anti rabbit IgG were from Santa Cruz Biotechnologies (Santa Cruz, CA, USA). D-[U- ^14^C]glucose, [1-^14^C]stearic acid, and [1-^14^C]sodium acetate were purchased from American Radiolabeled Chemicals, Inc (St. Louis, MO, USA). [Methyl-^3^H]thymidine, [2-^3^H]deoxyglucose and full-range rainbow molecular weight marker were from GE Healthcare Bio-Sciences Corp (Piscataway, NJ, USA). Lactate assay kit was from Eton Biosystems Inc (San Diego, CA, USA). BCA Bradford protein assay kit and super signal West pico chemiluminescent substrate were from Pierce (Rockford, IL, USA). Compound C was from Calbiochem (San Diego, CA, USA).

### Cell culture

WS-1, A549 and MCF-7 cells were cultured in MEM and H1299, H460 and MDA-MB-231 cells were grown in DMEM. Media was supplemented with 10% FBS, penicillin (100 U/ml), streptomycin (10 µg/ml), 1% non essential amino acids and 1% MEM vitamin solution (growing medium). Cells were grown at 37°C, 5% CO_2_, and 100% humidity.

### Cell models of SCD1 inhibition

A stable transfected clonal population of A549 cells bearing an antisense sequence of the human SCD1 gene (hSCDas) has been described previously [20]. In addition, pharmacological inhibition of SCD activity was assessed with novel chemical SCD inhibitor, CVT-11127 (N-(2-(6-(3,4-dichlorobenzylamino)-2-(4-methoxyphenyl)-3-oxopyrido[2,3-b]pyrazin-4(3H)-yl)ethyl) acetamide), whose synthesis and structure were described elsewhere [30]. CVT-11127 was used at concentrations in which the inhibition of SCD1 was greater than 95%. The total incubation time with the SCD inhibitor was, at least, 24 h in order to allow for one cell population doubling.

### SCD activity and de novo fatty acid synthesis

Δ9 desaturase activity in whole cells was determined as previously described [19]. Briefly, subconfluent cell monolayers were incubated with the specified concentration of SCD inhibitor or DMSO vehicle in the growing media for 24 h. Six hours prior harvesting, the cells were pulsed with [^14^C]stearic acid (0.25 µCi/60 mm petri dish) in culture medium containing 0.5% bovine serum albumin. Total cellular lipids were extracted according to Bligh & Dyer [33] and transesterified with BF_3_ in methanol for 3 h at 64°C under nitrogen atmosphere. The methyl esters were separated by argentation thin layer chromatography (TLC) following the procedure described by Wilson and Sargent [34], using a solvent phase consisting of hexane:ethyl ether (90∶10, by vol). The radiolabeled stearic and oleic acids were detected with a Storm scanner (Molecular Dynamics) and its optical density quantified with Imagequant software. For de novo fatty acid synthesis, the cells were incubated for 6 to 24 h with [U-^14^C]glucose or for 24 h with [^14^C]sodium acetate in the presence or absence of the SCD inhibitor. Cellular lipids were extracted as described and the amount of [^14^C]tracer incorporated into lipids was normalized to cellular protein content of cells grown in parallel petri dishes. Aliquots of [^14^C]glucose-labeled cell lipids were esterified and the levels of radiolabeled SFA and MUFA were determined by TLC as described above.

### Determination of glucose uptake

Preconfluent H1299 cells were incubated with 1 µM CVT-11127 or vehicle in glucose-deficient DMEM for 24 h. Cell were then pulsed for 7 minutes with 0.5 µCi/dish [^3^H]deoxyglucose in DMEM containing 0.5% BSA, 25 µM HEPES and 100 µM glucose. Labeled medium was quickly removed and residual labeling on the monolayers was removed by three washes with ice-cold PBS. Total radioactivity of cell homogenates was counted in a scintillation counter and [^3^H]DPM were normalized to cell protein content.

### Total cellular fatty acid composition

Total cellular lipids from A549 and H460 cancer cells treated with 10 uM or 1 uM CVT-11127, respectively, or vehicle for 24 h were extracted as described above. Heptadecanoic acid (C17:0) was added as internal standard at the beginning of the lipid extraction process. Transesterification and methylation of fatty acids from total lipids were performed as described by Lepage and Roy [35]. Fatty acid methyl ester composition was determined by gas chromatography using a Varian 3800 GC (Varian Inc, Palo Alto, CA), equipped with a DB-23 column (J&W Scientific Inc., Folsom, CA) and FID. Fatty acid methyl ester identification and response factors were determined using standard mixtures (NuChek Prep Inc., Elysian, MN). Chromatographic peaks were identified by comparison of their retention times with those of pure fatty acid standards and percent distribution was calculated.

### [^3^H]Thymidine incorporation into cell DNA

The rate of DNA synthesis was estimated by determining the levels of [^3^H]thymidine incorporation into DNA after pulsing the cells with the radiolabeled tracer for 2 h, followed by precipitation of total DNA and scintillation counting, as described [36]. Groups of cells were incubated with glucose free DMEM supplemented with different concentrations of glucose for 22 h prior the addition of the [^3^H]thymidine. In other experiments, the growing media was supplemented with 10 mM sodium pyruvate or sodium citrate for 22 h. For SCD inhibition, CVT-11127 was added at the indicated doses to the growing media for 22 h prior the labeling period. In all cases, the total incubation time with the metabolites or the inhibitor was 24 h.

### Determination of cell growth curves

The cells were seeded in 12 well plates (14,000 cells per well). Twenty four hours later, the monolayers were rinsed with PBS and groups of cells were incubated with 0.5 or 5.5 mM glucose in the growing media. The media was changed every 48 h thereafter. For some experiments, cells were incubated for 24 h and 48 h with increasing concentrations of sodium oleate up to 100 µM. Cellular proliferation was estimated by crystal violet staining following the procedure described by Menna et al. [37], with modifications. Briefly, cells were fixed with methanol, stained with 0.1% crystal violet in distilled water and rinsed three times with water. The dye in the stained cells was solubilized in 10% methanol, 5% acetic acid solution and quantified by spectrophotometry at 580 nm. The value of a blank well was subtracted in each case. The values at different time points were normalized to the data at 24 h after seeding to avoid differences due to disparity in cell adhesion efficiency or cell death.

### Lactate measurement

To determine the production of lactate, 9×10^4^ cells were seeded in 6 well plates and grown until monolayers reached 80% confluency. Cells were then rinsed with PBS and grown in 10%FBS, phenol-free MEM with the indicated concentrations of SCD inhibitor or vehicle for 24 h. For some determinations, the cells undergoing a blockade of SCD activity were incubated with 10 mM sodium citrate for 24 h. The content of lactate in the conditioned media was quantified with a lactate assay (Eton Biosciences Inc), according to the manufacturer's instructions and normalized to the total cellular protein.

### Immunoblotting

Preconfluent cells were treated as described, rinsed with ice cold PBS, scraped in cold hypotonic lysis buffer (20 mM Tris-HCl pH 7.5, 10 mM NaF, 1 mM EDTA, plus protease and phosphatase inhibitor cocktails) and sonicated. Fifty micrograms of total cellular proteins were resolved by SDS-PAGE and transferred onto a nitrocellulose membrane. After blocking, the membranes were incubated with polyclonal rabbit phospho-AMPKα (Thr172) and phospho-ACC (Ser79) overnight or monoclonal mouse anti β-actin for 2 h in 1∶1,000 dilutions. Horseradish peroxidase-conjugated secondary antibodies were used in 1∶10,000 dilutions. Proteins on the membrane were detected using a West pico chemiluminescence detection kit and quantified with a ChemiDoc (BioRad) digital image system using a QuantityOne software. All analyses of protein band density were done in the linear portion of the saturation curves and normalized to the β-actin content of the same samples.

### Determination of cellular protein

Total cellular protein content was measured by Bradford method, using BSA as a standard.

### Statistical analysis

Results from a representative experiment with at least 3 samples per experimental group are presented as means±S. D. Statistical significance of the data was determined by Student's t-test.

## Results

### Pharmacological inhibition of SCD activity impairs proliferation of cancer cells

**Figure 1:**
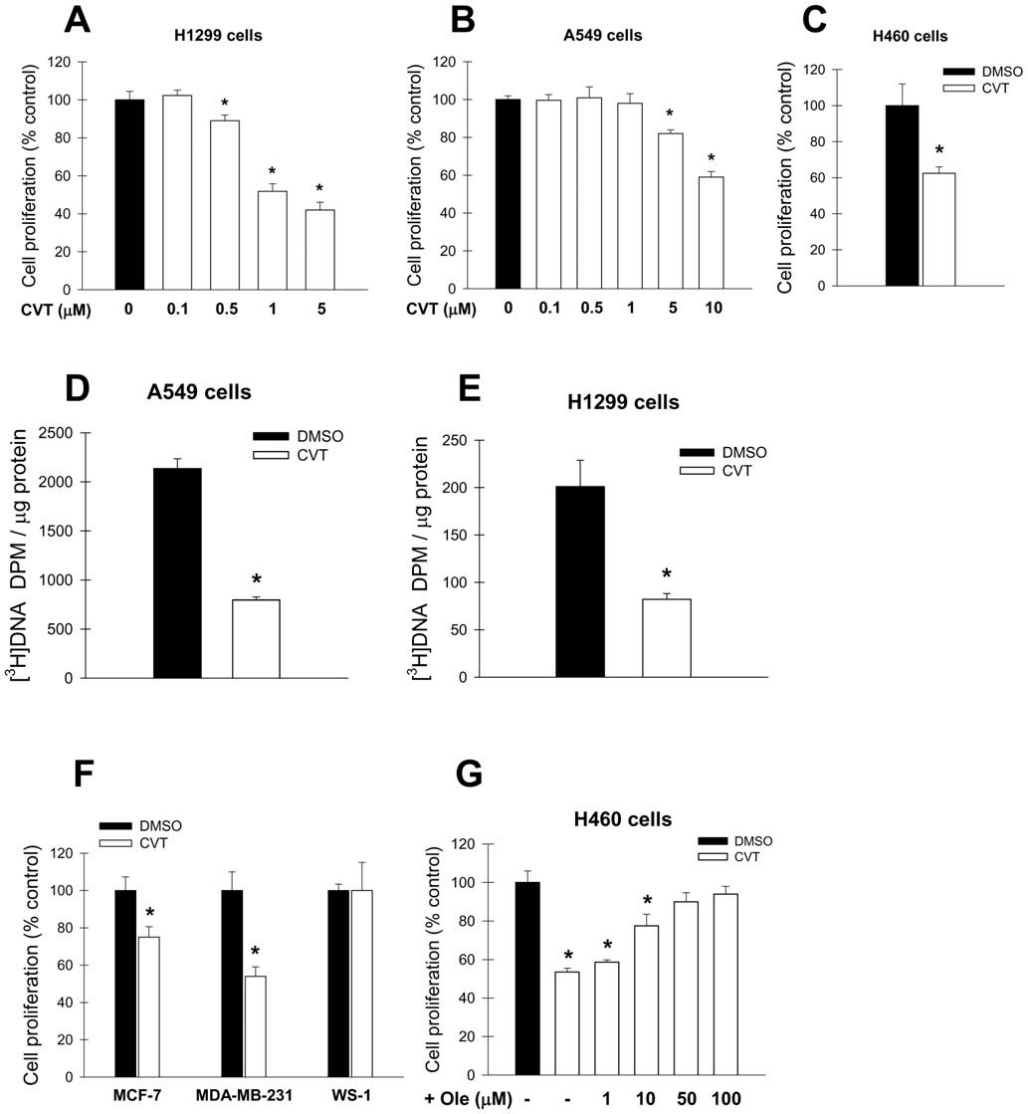
A novel small molecule inhibitor of SCD activity reduces cell proliferation in human lung and breast cancer cells. A549 (A) and H1299 (B) cells were incubated with with different concentrations of CVT-11127 (CVT) or DMSO vehicle for 24 h, as described, and cell proliferation was determined by Crystal violet assay. For a similar analysis, H460 cells (C) were treated with 1 µm CVT-11127 for 24 h. For the determination of DNA synthesis, A549 (D) and H460 (E) cells were incubated with 10 µM and 5 µM CVT-11127 or vehicle for 24 h and pulsed with [^3^H]thymidine (1 µCi/dish) for 2 h at 37°C. Total [^3^H]-labeled DNA was precipitated, radioactivity was quantified in a scintillation counter and normalized to protein concentration. F, MCF-7 and MDA-MB-231 breast cancer cells, and WS-1 human skin fibroblasts were incubated with 10 µM CVT-11127 (CVT) or DMSO vehicle for 24 h and cell proliferation was assessed by crystal violet staining method. E, H460 cells were incubated for 48 h with 1 µM CVT in presence of 1, 10, 50 and 100 µM sodium oleate complexed with BSA (1∶2 BSA:fatty acid ratio). Cell incubated with DMSO vehicle were considered the control group. Cell growth was estimated by crystal violet staining method. Values represent the mean±S.D. of triplicate determinations. *, p<0.05 or less vs control, by Student's t test.

We have previously reported that chronic depletion of SCD1 decreases the rate of cell proliferation in oncogene-transformed and cancer cells [19], [20]. To evaluate the potential use of newly developed SCD inhibitors as novel anticancer agents, we tested the potential growth inhibitory effect of CVT-11127, a novel small-molecule inhibitor of SCD activity, on several lung cancer cell lines. This compound was found to be an effective and desaturase-selective blocker of SCD activity in rat liver microsomal preparations and human HepG2 cells [30]. These authors reported that CVT-11127 does not inhibit the activity of rat microsomal Δ5 and Δ6 desaturases at concentrations up to 30 µM, indicating that CVT-11127 is selective for Δ9 desaturases. Thus, we incubated A549, H1299 and H460 cells with increasing concentrations of the SCD inhibitor for 24 h and found a progressive decrease in the rate of cell replication of cancer cells with respect to vehicle (DMSO)-treated cells (Figure 1). H1299 cells showed ∼55% and 65% decrease in cell proliferation rate in presence of 1 µM and 5 µM CVT-11127, respectively (Figure 1A). A549 cells were less sensitive to the cell growth inhibitory effect of CVT-11127, since these cells reduced their replication rate by 20% and 40% when treated with 5 µM and 10 µM CVT-11127, respectively (Figure 1B). Moreover, the proliferation rate of H460 cells treated with CVT-11127 was 60% lower than vehicle-treated controls (Figure 1C), indicating that these cells are as sensitive to the antigrowth effect of the SCD inhibitor as H1299 cells.

The incubation with the SCD inhibitor also resulted in a profound reduction (∼60%) in the incorporation of [^3^H]thymidine into newly synthesized DNA, an early marker of cellular proliferation rate, in both A549 and H1299 cancer cell lines (Figure 1D and E). In order to verify that the antigrowth effect of the SCD chemical inhibitor was not cell type-specific, MCF-7 and MDA-MB-231 breast cancer cells, as well as in normal human WS-1 fibroblasts, were incubated with CVT-11127 for 24 h and cell proliferation was assessed by Crystal violet staining (Figure 1F). It was found that the breast cancer cells were similarly sensitive to the cytostatic action of the small molecule SCD inhibitor. However, the proliferation of WS-1 normal skin fibroblasts was not affected by the treatment, suggesting that the antigrowth effect of SCD blockade may be dependent on the rate of cell replication. Furthermore, increasing concentrations of oleate in cell culture medium partially or totally reversed the inhibition of cell proliferation in H460 cells incubated with the small molecule SCD inhibitor (Figure 1G). Similar results were obtained with H1299 cells (data not shown). These findings indicate that oleic acid is essentially required for the fully active replication of cancer cells.

**Figure 2:**
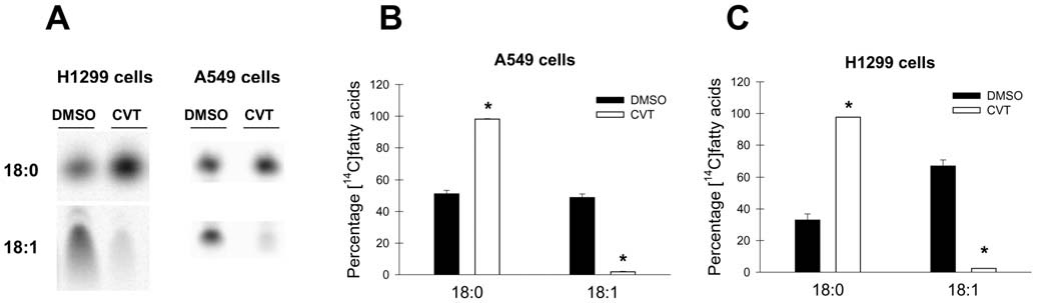
Specific inhibition of SCD activity by CVT-11127 compound. For the determination of Δ9-desaturating activity in cancer cells, A549 (A, B) and H1299 cells (A, C) were treated for 24 h with 10 µM and 5 µM CVT respectively, or DMSO vehicle. Six hours before harvesting, the cells were pulsed with [^14^C]18:0 (0.25 µCi/dish). After conversion into methylesters, fatty acids were separated on silver nitrate-impregnated TLC plates. The radioactive spots corresponding to SCD substrate and product ([^14^C]18∶1), were visualized with a Phosphor Imager (A) and quantified by densitometric analysis (B and C). Values represent the mean±S.D. of triplicate determinations. *, p<0.05, by Student's t test.

As expected, the growth inhibitory action of CVT-11127 was positively correlated with a significant inhibition of SCD activity in the lung cancer cells. As shown in Figure 2A–C, the treatment of A549 and H1299 cells for 24 h with 10 µM and 5 µM of CVT-11127, respectively, reduced SCD activity more than 95%, as assayed by the production of [^14^C]oleic acid from its radiolabeled precursor, stearic acid. This confirms that CVT-11127 is highly effective at suppressing the very high SCD activity found in these cancer cell lines.

**Figure 3:**
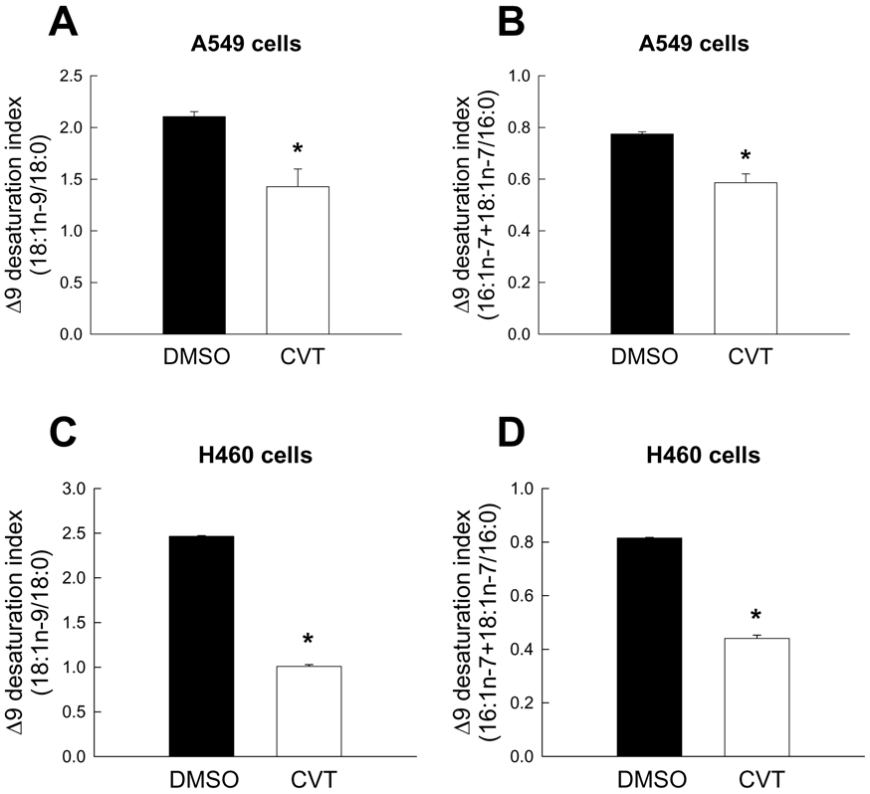
Decreased MUFA/SFA ratio in total lipids of lung cancer cells treated with a small molecule inhibitor of SCD activity. A549 cells (A, B) and H460 cells (C, D) were incubated with 10 µM and 1 µM CVT-11127 (CVT), respectively, or DMSO for 24 h. Cellular lipids were extracted and fatty acids were converted to their methylester form by transesterification as described in Materials and Methods. Fatty acid methyl ester composition was assessed by gas chromatography and percent distribution of fatty acids was calculated. Values express the ratio 18:1n-9/18:0 (A, C) and 16∶1-n7+18:1n-7/16:0 (B, D), and represent the mean±S.D. of 4–5 samples. *, p<0.01 or less, by Student's t test.

As a result of the reduction of SCD1 activity by the small molecule inhibitor, the distribution of SFA and MUFA in total cellular lipids of A549 cells was considerably altered (Figure 3A and B). The ratios MUFA to SFA in both n-7 and n-9 fatty acid series was decreased by 25% and 35%, respectively, in cells treated for 24 h with CVT-11127 with respect to vehicle-treated controls. In addition, the ratios MUFA/SFA in H460 cells were found diminished by 47% (n-7MUFA/SFA) and 60% (n-9MUFA/SFA) in cells undergoing a similar treatment with CVT-11127 with respect to DMSO-treated cells (Figure 3C and D). Perturbations in the cell MUFA content by incubations with the small molecule inhibitor were observed as early as 1 h after treatment (data not shown). These observations clearly show that the total fatty acyl chain composition of lipids in cancer cells is under the control of SCD1 activity, even when these cells were cultivated in medium with FBS, which contains significant amounts of MUFA.

### Inhibition of SCD1 decreases de novo lipid synthesis from glucose

**Figure 4:**
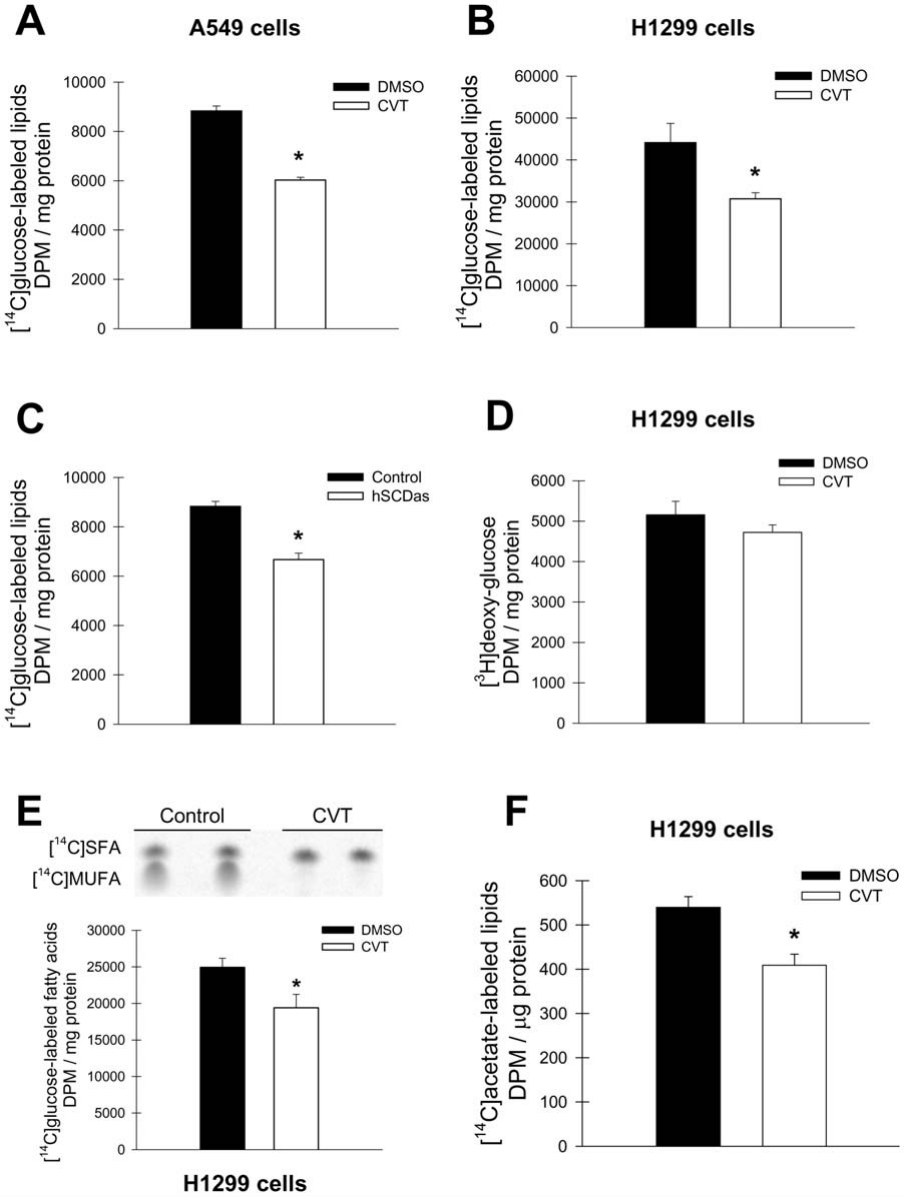
de novo lipid synthesis from glucose is impaired upon SCD inhibition. A549 cells (A) and H1299 cells (B) were incubated with 10 µM and 1 µM CVT-11127 (CVT), respectively, or DMSO for 24 h. Cells were then pulsed with 1 µCi D-[U-^14^C]glucose for up to 24 h. C, A549 cells with a stable knockdown in SCD1 expression (hSCDas) and mock-transfected control cells were subjected to a similar incubation with [^14^C]glucose. Cellular lipids were extracted and incorporation of [^14^C]glucose into total lipids was quantified by scintillation counting and normalized to protein concentration. D, basal glucose uptake was assayed in H1299 cells treated with 1 µM CVT or vehicle for 24 h by estimating the uptake of [^3^H]deoxyglucose. E, H1299 cells were incubated with [^14^C]glucose in presence or absence of 1 µM CVT for 6 h and levels of total [^14^C]fatty acids, as well as radiolabeled SFA and MUFA (upper panel), were determined by argentation TLC as described in Materials and methods. F, the rate of lipid synthesis in H1299 cells was assessed by incubation with 1 µM CVT or vehicle and 0.5 µCi/dish of [^14^C]acetate for 24 h. Lipids were extracted and radioactivity of total lipids was determined by scintillation counting. Values represent the mean±S.D. of triplicate determinations. *, p<0.05 or less, by Student's t test.

Cancer cells have modified a set of signaling and metabolic pathways to enhance the use of glucose as main substrate for macromolecular biosynthesis and for energy-generating reactions [38]. Previously, we showed that chronic depletion of SCD1 suppresses the overall rate of lipogenesis [19], [20]. In order to dissect the mechanisms of metabolic regulation by SCD1, we investigated the effect of acute inhibition of SCD activity with the novel small molecule CVT-11127 on the lipogenic pathways. To document the effect of acute SCD inactivation on glucose-mediated lipid biosynthesis, A549 and H1299 cells were treated with SCD inhibitor or vehicle for 6 h and 24 h respectively, and the formation of total cellular lipids from [^14^C]glucose was determined. As displayed in Figure 4A and B, the incorporation of radiolabeled glucose into total cellular lipids was significantly impaired (30%) in SCD inhibitor-treated H1299 and A549 cells with respect to vehicle-treated controls. As previously observed [20], the incorporation of radiolabeled glucose into total lipids decreased by ∼20% in stable SCD1-knockdown A549 (hSCDas) cells (Figure 4C), confirming that the presence of a fully active SCD1 is crucial for sustaining the accelerated glucose-mediated lipogenesis in cancer cells. The alteration in the formation of [^14^C]glucose-labeled lipids was not caused by a deficient uptake of glucose because we observed no changes in the rate of [^3^H]deoxyglucose uptake in cells treated with CVT or vehicle (Figure 4D). Incorporation of radiolabeled glucose into the fatty acids of cancer cell lipids was reduced by treatment with CVT (Figure 4E), suggesting that the abnormal formation of lipids in cells undergoing inhibition of SCD1 may be caused by a defective de novo fatty acid biosynthesis. As expected, the production of glucose-labeled MUFA was almost fully suppressed in H1299 cells with a block in SCD activity (Figure 4E , upper panel). Furthermore, the biochemical alteration in lipogenesis in cells with chemical blockade of SCD appears to be located downstream the formation of acetylCoA since a reduced formation of [^14^C]acetate-labeled lipids was observed in CVT-treated H1299 cells with respect to vehicle-treated controls (Figure 4F).

### Stimulation of glycolysis/lipogenesis does not reverse the decreased proliferation of SCD1-deficient cells

**Figure 5:**
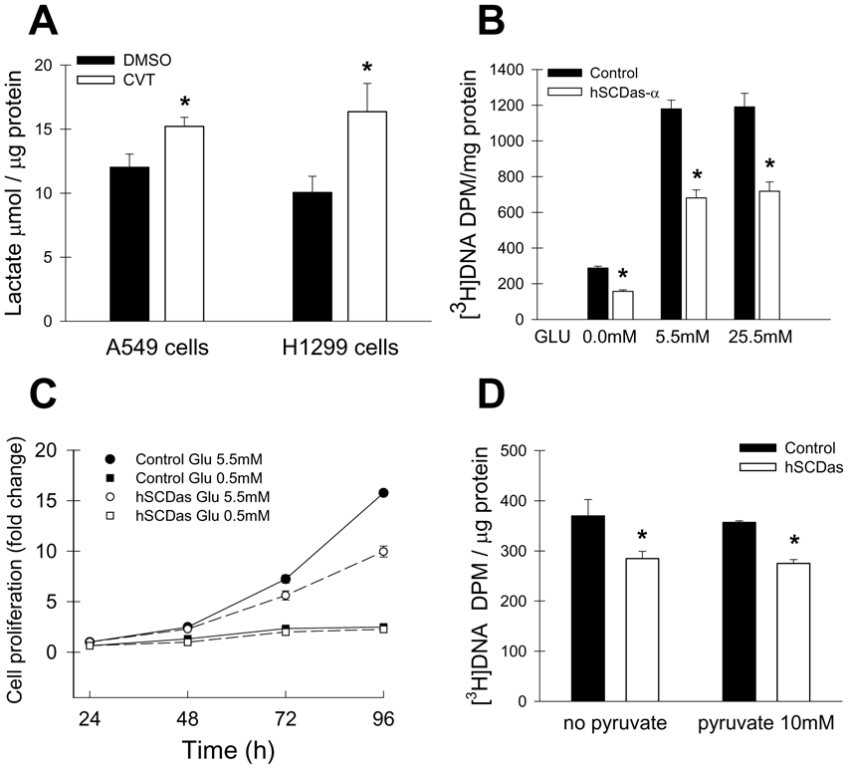
Induction of glycolysis/lipogenesis does not rescue the reduced proliferation of cells with depleted SCD. A, A549 and H1299 cells were incubated with 10 µM and 5 µM CVT-11127 (CVT) respectively, or vehicle (DMSO) in phenol red-free media for 24 h. The lactate concentration in the conditioned media was determined with a colorimetric kit as described under Experimental procedures and normalized to cellular protein. Control and SCD1-deficient (hSCDas) A549 cells were incubated with the indicated concentrations of glucose (GLU) (B) or pyruvate (C) for 24 h and pulsed with [^3^H]thymidine (1 µCi/dish) for 2 h at 37°C. Total [^3^H]-labeled DNA was precipitated, radioactivity was quantified in a scintillation counter and normalized to protein concentration. D, growth curve of control and hSCDas A549 cells in media containing either 0.5 or 5.5 mM glucose. Cells were seeded in 12 well plates (14,000 cell/well) and after 24 h regular growing media was switched to glucose-supplemented media. Media were replaced with fresh glucose-supplemented media every 48 h thereafter. At the indicated times, cell population was determined by crystal violet staining as described under Experimental procedures. Values represent the mean±S.D. of triplicate determinations. *, p<0.05, by Student's t test.

As described above, both acute pharmacological blockade of SCD activity and stable gene knockdown of SCD1 significantly alter the rates of glucose-mediated lipogenesis and cell replication. We hypothesized that a perturbation in aerobic glycolysis could be the primary cause of the impaired proliferation and survival of SCD1-deficient cells ([19], [20] and Figure 1). We then determined the rate of glycolysis in A549 and H1299 cells by assessing the levels of lactate in the conditioned media, an indicator of aerobic glycolytic rate in cancer cells [39], and found an increase of 30–60% in cells undergoing pharmacological inhibition of SCD compared with vehicle-treated controls (Figure 5A). To confirm that a change in the flux of glycolytic metabolites was not responsible for the deficient replication of SCD1-ablated cells, we incubated the cells in medium containing very low (0.5 mM), normal (5.5 mM) or high (25 mM) levels of glucose and determined the rate of DNA synthesis and cell growth. As shown for other cancer cell lines, A549 cells displayed strict glucose dependence for proliferation (Figure 5B). When grown in essentially glucose-free MEM (containing ∼0.5 mM glucose from the 10% FBS-supplementation) for 24 h, the incorporation of radiolabeled thymidine into the DNA of SCD1-deficient (hSCDas) and control cells was reduced by 70% when compared to cells growing under standard culture conditions (5.5 mM glucose). However, the significantly decreased rate of DNA synthesis observed in SCD1-ablated cells persisted regardless of the glucose level in the culture media. These differences in cell growth rate between SCD1-deficient cells and controls, grown in both low and normal glucose-containing media, were maintained over a course of 96 h incubation (Figure 5C). Furthermore, not even the presence of 25.5 mM glucose in the growth media was able to reverse the reduced rate of DNA synthesis of SCD1 deficient cells, suggesting that the abnormally low rate of replication of cells with reduced SCD1 can not be attributed to an altered utilization of glucose. Alternatively, we incubated the hSCDas and control cells with 10 mM pyruvate, the end product of glycolysis and fuel for the tricarboxylic acid cycle, for 24 h and examine DNA formation. As shown in Figure 5D, pyruvate was ineffective for inducing DNA synthesis in either cell group, or restoring the impaired proliferation rate of SCD1-ablated cells to control levels. This finding further confirms that the alteration in lipogenesis and cell growth caused by the inhibition of SCD1 can not be ascribed to deficient production of glycolytic products.

### Inhibition of SCD1 induces AMPK and reduces ACC activity

**Figure 6:**
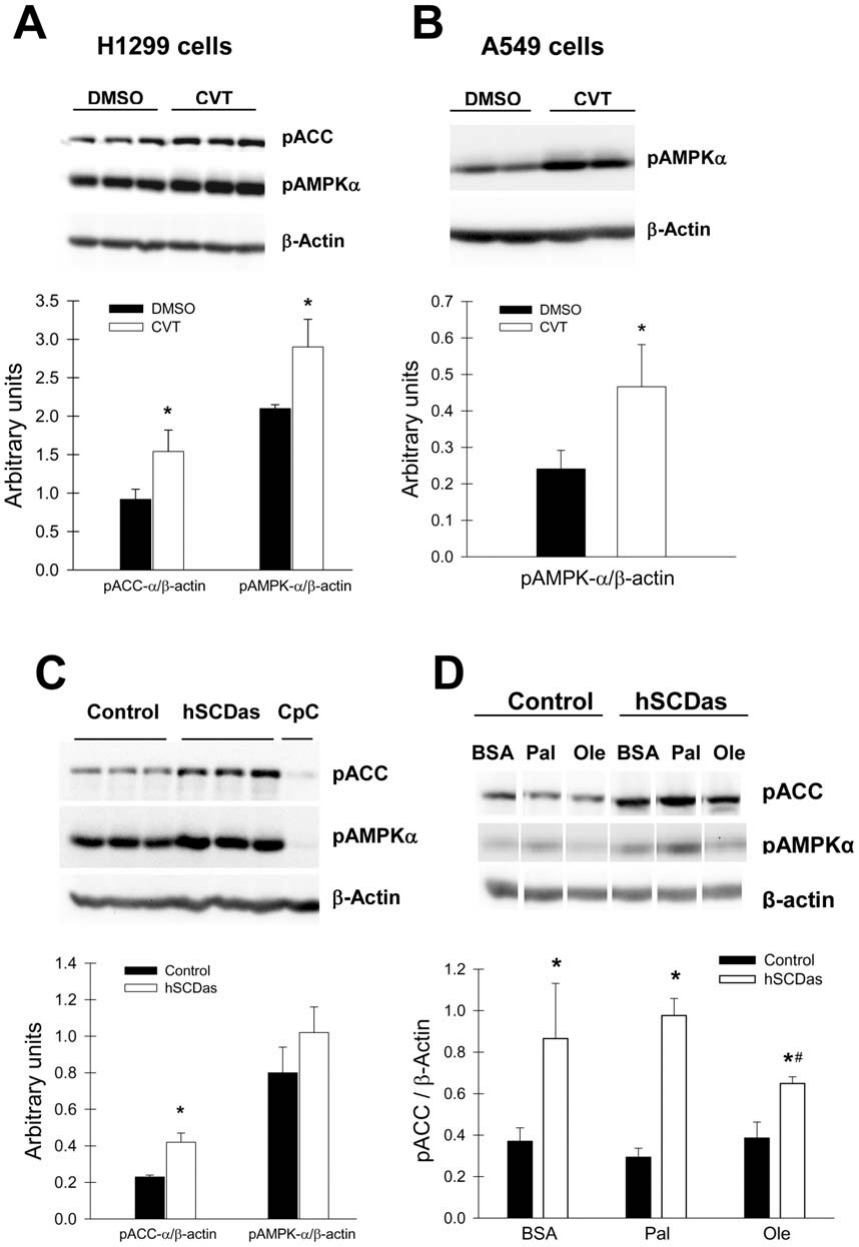
Inhibition of SCD1 upregulates AMPK phosphorylation and activity. A549 and H1299 cells were incubated with 10 µM and 5 µM CVT-11127 (CVT) respectively, or DMSO for 24 h. The levels of phospho-ACC (Ser79) and phospho-AMPKα (Thr172) were determined by Western Blot in CVT-11127-treated cells (A–B) and SCD1-deficient A549 cells (C) and normalized to β-actin content. Cells treated with 20 µM Compound C (CpC) were included as a control. D, regulation of ACC activity by fatty acids. SCD1-deficient (hSCDas) and control A549 cells were serum-starved (0.1% FBS) for 24 h and incubated for 30 min in serum-depleted media with or without 100 µM palmitic (Pal) or oleic (Ole) acid complexed with 0.5% w/v fatty acid-free bovine serum albumin (BSA). Cellular levels of phospho-ACC (Ser79), phospho-AMPK and β-actin were determined by Western blot. Values represent the mean±S.D. of triplicate determinations. *, p<0.05 or less, by Student's t test.

Cellular growth and proliferation depends on the coordinated and opposed regulation of anabolic and catabolic pathways. AMPK, a central cell fuel sensor, is activated upon energy deficiency and by other conditions of cellular stress [25]. One of the main metabolic targets of the AMPK pathway is ACC. Phosphorylation of ACC by AMPK reduces its activity with the consequent decrease in de novo fatty acid synthesis. We therefore determined whether the activation of these catabolic signals could account for the reduced lipogenesis in our cellular models of SCD1 deficiency. Initially, we assessed the effect of the pharmacological SCD inhibitor CVT-11127 on AMPK activation in H1299 and A549 cells. After 24 h treatment, the levels of the phosphorylated AMPK α-subunit and ACC were analyzed by immunoblotting. As shown in Figure 6A and B, and in agreement with the observed decrease in lipogenesis, the blockade of SCD activity resulted in increased phospho-AMPK in both cancer cell lines as well as in an increase in phospho-ACC in H1299 cells treated with the SCD inhibitor. Furthermore, in A549 cells with the stable knockdown of SCD1 expression, phosphorylation of ACC-α was greater that in mock-transfected control cells (Figure 6C). As positive control for the downregulation of AMPK in this experiment, some cells were treated with compound C, a well known blocker of AMPK phosphorylation, which showed a notable reduction on phospho-ACC. Morover, the phosphorylation of ACC was also significantly augmented (∼80%) in hSCDas cells, indicating that AMPK activity was effectively induced in a condition of SCD1 deficiency. Incubation of SCD1-deficient cells with palmitic acid did not significantly change the phosphorylation of either enzymes, however, oleic acid significantly reduced the levels of phosphor-ACC (Figure 6D). Overall, these results suggest that the inhibition of SCD1 suppresses lipogenesis by the induction of AMPK pathway and the consequent inactivation of ACC.

### Is activation of AMPK pathway upon SCD1 inhibition a protective mechanism in cancer cells?

**Figure 7:**
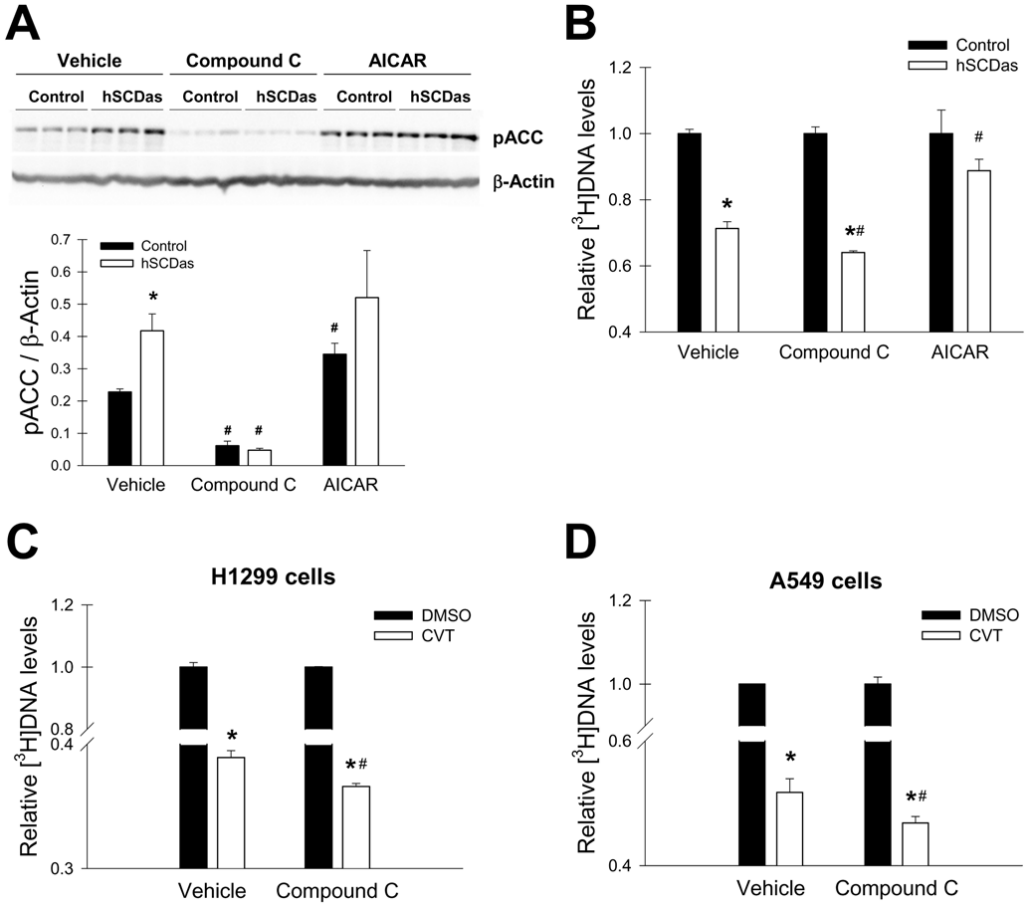
Inhibition of AMPK further reduces cellular proliferation in cells with a blockade in SCD1. *A*, control and SCD1-deficient A549 cells were incubated with compound C (20 µM), AICAR (0.25 mM) or vehicle for 24 h and the levels of phospho-ACC (Ser79) were estimated by Western Blot. Protein bands were quantified by densitometric analysis and normalized to β-actin content. hSCDas and mock-transfected control cells (*B*), or CVT-11127-treated (CVT) H1299 (*C*) and A549 (*D*) cells were treated with Compound C, AICAR or vehicle and pulsed with [^3^H]thymidine for 2 h. The radiolabeled DNA was quantified as described in Materials and methods. Results are expressed as fold-change in total [^3^H]DNA levels over vehicle-treated control. Values represent the mean±S.D. of triplicate determinations. *, p<0.05 vs DMSO under the same conditions; #, p<0.05 vs CVT treated cells incubated with vehicle by Student's t test.

Although high rates of de novo fatty acid synthesis are necessary for active cellular proliferation [40], the accumulation of SFA has been shown to be deleterious for both normal and cancer cells [19], [41]–[44]. We have previously reported that SCD1-deficient cells display an increased content of SFA in all major lipid species, including the non-esterified fatty acid fraction, a condition that triggers the program of cell death [19], [41]. Thus, we speculated that the activation of AMPK and the consequent inhibition of fatty acid synthesis could ultimately be a protective mechanism against the accumulation of SFA due to deficient SCD1 activity. Therefore, we examined the rate of DNA synthesis in cancer cells upon either inhibition or stimulation of AMPK activity. As expected, the blockade of AMPK activity by compound C translated into a notable reduction in ACC phosphorylation (Figure 7A). Treatment with compound C provoked a reduction in DNA synthesis in both control and SCDas cells, although this decrease was more profound in SCD1-deficient cells (Figure 7B). A similar depressing effect in the rate of DNA formation was also detected in CVT-11127-treated H1299 and A549 cells with compound C (Figure 7C–D). Conversely, AICAR, a well-studied pharmacological activator of AMPK, restored the impaired cellular proliferation of SCD1-deficient A549 cells to control values (Figure 7B). This set of studies suggests that the activation of AMPK may be part of a mechanism that shuts down fatty acid synthesis when conversion of SFA into MUFA is not appropriately operating.

**Figure 8:**
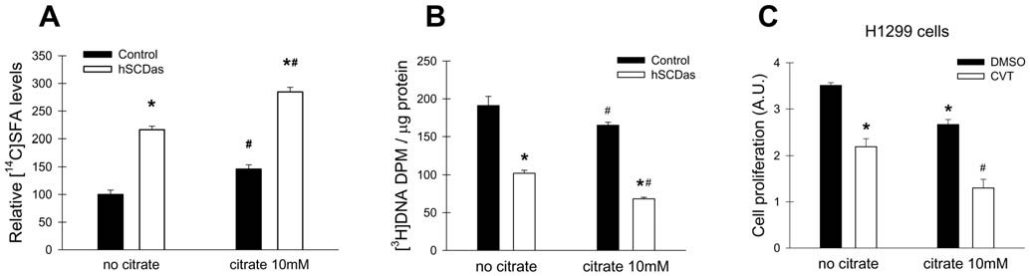
Citrate induces SFA synthesis and reduces proliferation in SCD1-deficient cells. A, Control and hSCDas cells were incubated with or without 10 mM sodium citrate for 24 h in the presence of 0.45 µCi/dish [1-^14^C]acetate. The total cellular lipids were extracted and transesterified as described under Materials and Methods. The radiolabeled SFA were resolved on silver nitrate-impregnated TLC plates, visualized with a Phosphor Imager and quantified by densitometric analysis. B, Control and hSCDas cells were incubated with or without 10 mM sodium citrate for 24 h and pulsed with [^3^H]thymidine for 2 h. The radiolabeled DNA was quantified as described. Values are the mean±S.D. of triplicate determinations. *, p<0.05 vs control; #, p<0.05 vs no citrate, by Student's t test. C, H1299 cells were incubated for 24 h with 5 µM CVT-11127 (CVT) or DMSO vehicle in growing media with or without 10 mM sodium citrate. Cellular proliferation was estimated by Crystal violet staining. Bars represent the mean±S.D. of triplicate determinations. *, p<0.05 vs DMSO; #, p<0.05 vs CVT without citrate, by Student's t test.

With the aim of testing whether this effect was due to the specific modulation of fatty acid synthesis, we incubated the cells with an excess of citrate (10 mM), a potent allosteric activator of ACC and a metabolite that provides cytosolic acetylCoA for de novo fatty acid synthesis. Incubations with citrate led to a significant increase in incorporation of [^14^C]acetate into fatty acids in both control and SCD deficient cells (data not shown), an effect likely due to an induction of ACC activity. The amount of [^14^C]SFA was further augmented upon induction of fatty acid synthesis by citrate in SCD1-ablated cells with respect to control A549 cells (Figure 8A) indicating citrate was able to overcome the block in fatty acid synthesis resulting from SCD deficiency. To assess a potential effect of increased SFA synthesis on the rate of mitogenesis we determined the levels of DNA synthesis in hSCDas and control cells in presence or absence of citrate (Figure 8B). In control cells, citrate treatment had minimal effect on cellular proliferation (0 to 15% reduction in thymidine incorporation), while in SCD1-deficient cells the decrease was ≥30% with respect to vehicle-treated cell counterparts. This demonstrates that while citrate can increase SFA production in the absence of SCD1 activity, the increased concentration of SFA is not beneficial and adds to the anti-proliferative effects of SCD1 deficiency. Additionally, we investigated the effect of 10 mM citrate supplementation on the replication of H1299 cells, treated with the SCD inhibitor or vehicle, for 24 h (Figure 8C). Again, a significant reduction in proliferation was observed in cells treated with citrate, CVT-11127 or both, however, the combination of citrate and the SCD inhibitor led to the most profound reduction in cell growth. An accumulation of cytosolic citrate could also hinder glycolysis by suppressing phosphofructokinase activity [45], thereby inhibiting cell growth. However, the decrease in cellular proliferation in SCD1-deficient cells was not due to a reduction in glycolysis since the production of lactate, a terminal product of glycolysis, was increased upon citrate treatment (data not shown), arguing in favor of a greater channeling of citrate towards SFA synthesis due to the activation of ACC.

## Discussion

In the present work, we demonstrate that SCD1, a key enzyme in the biosynthesis of MUFA, controls glucose-mediated lipogenesis by modulating the rate of synthesis of fatty acids, thereby providing cancer cells with the necessary lipid structures and signals to sustain their fast replication rate. The conversion of glucose into lipogenic substrates is a critical metabolic event in cancer cells [38]. The increasing demand of lipid metabolites for membrane production in cancer cells is met by the concerted upregulation of the enzymes of both glycolysis and de novo fatty acid biosynthetic pathways [9], [38], [40]. High activity levels of ATP-citrate lyase, ACC and FAS, which respectively catalyzes the sequential synthesis of acetylCoA, malonylCoA and palmitic acid, have been shown to be essential for cancer cell proliferation [4]–[9]. Previous reports from our laboratory [19], [20] and the present studies show that the subsequent conversion of endogenously synthesized SFA into MUFA by SCD1 is an essential step in cancer cell growth.

Our results reveal that SCD1 regulates de novo fatty acid synthesis in cancer cells by modulating ACC activity. The carboxylation of cytosolic acetylCoA to form malonylCoA by ACC is the committed step in de novo fatty acid synthesis [46]. As expected for a rate limiting enzyme, ACC is tightly regulated at multiple levels [47]. Although we can not completely rule out some degree of transcriptional regulation of ACC in conditions of reduced SCD1, especially in the stable SCD1-knockdown cells, its long half-life (∼3 days) [48] suggests that a different regulatory mechanism is operating under SCD1 inhibition. At least two interactive posttranslational modifications, polymer-monomer transition and reversible phosphorylation, determine the rate of ACC activity. ACC exists as either active polymer or inactive monomers [49]. AcylCoAs are potent allosteric inhibitors of ACC, inducing its depolymerization [27]–[29]. The most potent inhibitors are saturated fatty acyl-CoAs with 16–20 carbons at <1 to 6.5 nM concentrations. Therefore, the elevated intracellular levels of SFA in cells with reduced SCD1 [19], [20] may promote the depolymerization and the inactivation of ACC, and consequently, the downregulation of lipid synthesis.

Our data also suggest that SCD1 activity may contribute to the enhancement of lipid biosynthetic reactions in cancer cells by inactivating catabolic regulators such as AMPK. In conditions of low cellular energy or metabolic stress, AMPK is activated by phosphorylation of its α-subunit [50]. A main metabolic target for AMPK is ACC. Phosphorylation of serine residues in ACC by active AMPK inhibits its catalytical activity by both decreasing its V_max_ and increasing the Ka for citrate its allosteric activator [51], [52]. Both stable SCD1 gene knockdown and acute pharmacological inhibition of SCD induced AMPK activation and the subsequent phosphorylation of ACC in lung cancer cells, suggesting a second mechanism for the inhibition of fatty acid synthesis and lipogenesis observed in SCD1-deficient cells [19], [20]. Interestingly, SCD1 knockout mice exhibit increased AMPK activity in liver and muscle [53], [54], indicating that an SCD1-mediated regulation of AMPK operates in human and mouse tissues. Furthermore, the activation of AMPK may be responsible for other antilipogenic effects of SCD1 ablation in neoplastic human cells, such as the decreased synthesis of cholesterol [19]. In this regard, it has been reported that AMPK phosphorylates and inactivates HMG-CoA reductase a critical enzyme in cholesterol synthesis [26].

The mechanisms by which SCD1 regulates AMPK are currently unknown. Our results rule out a relevant role for LKB1, one of the protein kinases that modulate AMPK activation, since A549 cells do not express active LKB1 whereas H1299 cells display an active form of this tumor suppressor [55]. Moreover, although there is some discrepancy in the literature [56], [57], increased cellular acylCoAs, specially palmitoylCoA, a key substrate for SCD1 and likely elevated in SCD1 deficient cells [19], was linked to greater levels of catalytically active AMPK in several tissues [26], [58]–[60]. Our observation that oleic acid induces a dephosphorylation of ACC further reinforces the arguments in favor of a role of MUFA on ACC activation.

A remarkable biological effect of the stable depletion of SCD1 is the suppression of the malignant phenotype of neoplastic cells, characterized by a dramatic reduction in cell proliferation and in vitro invasiveness [19], the activation of programmed cell death [19], [21] and a reduction in the tumorigenic capacity [20]. Here we observed that the acute inhibition of SCD (∼95% reduction after 24 h treatment) with a specific small molecule inhibitor drastically reduced the rate of proliferation of a variety of human lung and breast cancer cell lines. Since the activation status of p53, pRb, and LKB1 as well as other oncogenes and tumor suppressors varies among these cell lines, the consistent decrease in the rate of proliferation of SCD1-deficient cells suggests that SCD1 is involved in a crucial metabolic step that is common to many cancer cell types. This finding also provides compelling evidence that SCD inhibitors may have a future role in the treatment of some cancers.

The aforementioned perturbations in the biological phenotype of cancer cells induced by a blockade of SCD1 are likely caused, at least in part, by a deficient production of membrane-building molecules such as phospholipids and cholesterol to support the continued proliferation of these cells [19], [20]. Active biosynthesis of lipids is required not only for sustaining continue mitogenesis [41], [61] but is also necessary for avoiding the entry into the program of apoptosis [62]. Although glucose-mediated lipogenesis was severely affected by SCD1 inhibition, several lines of evidence suggest that the abnormally low cell proliferation rate in SCD1-ablated cells was not caused by a deficit in the availability of glucose or the glycolysis-derived products. Supraphysiological concentrations of glucose or pyruvate (the final product of glycolysis) could not restore growth. In addition, AMPK, which was activated in slowly proliferating SCD1-deficient cells, is known to upregulate glucose uptake and glycolysis [25]. Altogether, these results suggest that in cells with reduced SCD1 activity the decreased de novo synthesis of lipids, as well as the parallel suppression of cell growth, were not due to the deficient production of pyruvate from glycolysis.

In our SCD1-deficient cellular models, we observed that fatty acid synthesis was downregulated, suggesting that this may be an adaptive inhibitory metabolic response to limit the potential harm of excess SFA accumulation when the conversion of SFA to MUFA is blocked. Several findings support this antilipotoxic mechanism. For instance, forcing fatty acid synthesis with citrate produced a greater increase in the formation of SFA in SCD1-deficient when compared to normal cells and a more profound decrease in cell proliferation. In addition, exogenously added citrate enhanced the effect of the SCD inhibitor on the proliferation of A549 and H1299 cells. In line with these observations, we have previously shown that SCD1-deficient cells are more sensitive to the induction of apoptosis by exogenously added SFA [19]. Other experimental conditions employed in our experiments to activate lipogenesis such as the pharmacological inhibition of AMPK further depressed the low proliferation rate of cancer cells with reduced levels of SCD1. Remarkably, the pharmacological activation of AMPK rescued the cellular proliferation in SCD1-ablated cells. A protective role of AMPK activation against the accumulation of SFA and consequent cytotoxicity has also been reported in normal cells, including astrocytes, pancreatic β-cells, and muscle [63]–[65], suggesting that a block in fatty acid biosynthesis when SFA are abundant may represent a ubiquitous safeguard mechanism.

**Figure 9:**
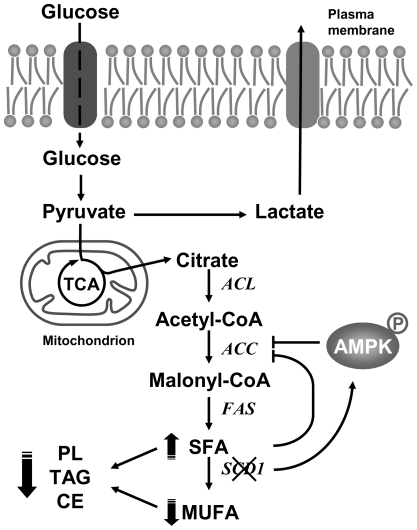
Hypothetical mechanism of regulation of lipid synthesis by SCD1 in human cancer cells. ACC, acetylCoA carboxylase; ACL, ATP citrate lyase; AMPK, AMP-activated protein kinase; CE, cholesterol esters; FAS, Fatty acid synthase; PL, phospholipids, SCD1, Stearoyl-CoA Desaturase 1; TAG, triacylglycerols.

We have previously reported that stable knockdown in SCD1 gene expression reduces Akt activity in cancer cells [20], suggesting an overall shift towards cellular catabolism, which is incompatible with cellular growth and proliferation. Here, we have shown that genetic and pharmacological inhibition of SCD1 triggers the activation of AMPK and impairs de novo fatty acid synthesis from glucose. Based on current results and previous data [19], [20], we postulate that by controlling the levels of SFA through conversion into MUFA, SCD1 modulates the rate of fatty acid synthesis and consequently the overall biosynthesis of glycerolipids (Figure 9). SCD1 regulates the biosynthesis of fatty acids by at least two mechanisms: 1) the regulation of the cellular content of palmitic acid, which is a powerful negative regulator of ACC activity [49], and 2) by controlling the phosphorylation status and hence the activation of AMPK, which in turn phosphorylates ACC reducing the enzyme activity and the overall rate of lipogenesis in cells. Additionally, SCD1 may prevent the harmful effects of excess SFA that result from constitutively active fatty acid synthesis in cancer cells [40]. Finally, by converting the excess SFA into MUFA, especially oleic acid, SCD1 may be able to enhance the activity of several signaling pathways, such as Akt and PKC, that are activated by oleate [43], [66], [67].

In conclusion, we have added evidence that SCD1 activity can modulate lipogenesis and the signaling pathways that control metabolism in cancer cells. This results in SCD1 playing a major role in cancer cell proliferation and survival as well as in tumor formation and progression. From a clinical perspective, inhibiting or ablating SCD1 may represent a promising therapeutic approach for treating deadly and widespread forms of cancer such as lung and breast cancers.
